# Genome-Wide Identification of the *KNOX* Gene Family in Japanese Apricot (*Prunus mume* Sieb. et Zucc.) and Functional Characterization of *PmKNAT2* Genes

**DOI:** 10.3390/genes14040939

**Published:** 2023-04-18

**Authors:** Yang Bai, Ting Shi, Xiao Huang, Pengyu Zhou, Kenneth Omondi Ouma, Zhaojun Ni, Feng Gao, Wei Tan, Chengdong Ma, Yufan Ma, Zhihong Gao

**Affiliations:** College of Horticulture, Nanjing Agricultural University, Nanjing 210095, China

**Keywords:** Japanese apricot, *PmKNAT2a*, *PmKNAT2b*, phylogenetic analysis, gene expression, lignin

## Abstract

The *Knotted1-like Homeobox* gene is crucial for plant morphological development and growth. Physicochemical characteristics, phylogenetic relationships, chromosomal localization, cis-acting elements, and tissue-specific expression patterns of the 11 *PmKNOX* genes found in the Japanese apricot genome in this study were examined. Proteins of 11 *PmKNOX* were soluble proteins with isoelectric points between 4.29 and 6.53, molecular masses between 15.732 and 44.011 kDa, and amino acid counts between 140 and 430. The identified *PmKNOX* gene family was split into three subfamilies by jointly constructing the phylogenetic tree of KNOX proteins in Japanese apricot and *Arabidopsis thaliana*. Combined outcomes of the analyzed conserved motifs and gene structures of the 11 *PmKNOX* genes from the same subfamily displayed comparable gene structure and motif patterns. The 11 *PmKNOX* members were distributed across six chromosomes, while two sets of *PmKNOX* genes were found to be collinear. Analysis of the 2000 bp promoter upstream of the coding region of the *PmKNOX* gene revealed that most *PmKNOX* genes might be involved in the physiological metabolism, growth and development processes of plants. The *PmKNOX* gene expression profile revealed that these genes were expressed at varying levels in different tissues, and most of them were linked to the meristems of leaf and flower buds, suggesting that *PmKNOX* may be involved in plants’ apical meristems. In *Arabidopsis thaliana*, functional validation of *PmKNAT2a* and *PmKNAT2b* revealed that these two genes might be involved in regulating leaf and stem development. In addition to laying the groundwork for future research on the function of these genes, understanding the evolutionary relationships between members of the *PmKNOX* gene family provides opportunities for future breeding in Japanese apricots.

## 1. Introduction

The *Knotted1-like Homeobox* genes are a class of transcriptional regulators consisting of homologous heterotypic cassettes that bind to target genes’ cis-regulatory regions and play complex regulatory roles in eukaryotic individual development and cell differentiation. The first *Homeobox gene* isolated in plants was the *KNOTTED1* (*ZmKN 1* or *KN1*) gene of maize (*Zea mays*), which maintains the differentiation of the shoot apical meristem (SAM) and is essential for plant growth and development [1,2]. Subsequently, *KNOTTED1-like* genes were cloned in *Arabidopsis*, rice (*Oryza sativa*), barley (*Hordeum vulgare*), tomato (*Solanum lycopersicum*) and other plant species, and phylogenetic analysis revealed that these genes belong to the same gene family, known as the KNOX (KNOTTED I-like homeobox).

The KNOX gene family is classified into classes I, II and M based on structural features, phylogenetic relationships, and expression patterns in many plant species [3,4,5,6]. KNOX consists of four domains: KNOX1, KNOX2 at the conserved N-terminal region, and the ELK domain and homeodomain (HD) at the conserved C-terminal region. Class I *KNOX* genes have distinct spatiotemporal expression patterns and both redundant and distinct functions. The class I *KNOX* genes are most similar to *Kn1* and are predominantly expressed in the SAM overlap region of the apical meristem, where it regulates cell differentiation and ultimately influences the morphogenesis of lateral organs [7]. The class II *KNOX* genes form a monophyletic group and are expressed in all tissues of the plant. However, the mutants lack a distinct phenotype. Among them, the *KNAT3* gene is associated with the abscisic acid response process during seed germination and seedling growth and development in *Arabidopsis* [8], the *KNAT7* gene is associated with secondary plant cell wall synthesis [9], and deletion of the *KNAT7* gene in rice results in thicker cell wall and larger seeds [10]. *KNATM* is a new class of KNOX family genes that encodes a MEINOX structural domain as opposed to a homeodomain [11]. *KNATM* was found to be mainly expressed in the proximal domains of plant organ primordia and the borders of mature organs; it may be involved in sensing or generating flowering signals. Since the class I *KNOX* genes are actively involved in plant growth and the developmental [12], the focus of current studies is on this class of *KNOX* genes.

The class I KNOX genes regulate the growth and development of lateral organs, are expressed in apical meristematic tissues and determine the cell fate of meristematic tissue. KNOX proteins may serve as general orchestrators of growth-regulator homeostasis at the shoot apex of *Arabidopsis* by activating cytokinin (CK) and inhibiting gibberellin (GA) production concurrently. Consequently, increasing meristem activity [13]. Overexpression of class I *KNOX* genes results in altered patterns of plant leaf development, such as the appearance of serrated cut leaves in *Arabidopsis* when *BP* is ectopically expressed, whereas transfer of the maize *KN1* gene into *Arabidopsis* results in the observation of distinct clefts observed at the leaf margin [14]. The *KNOX* genes also play a regulatory role in the plant’s apical meristem, the layer responsible for vascular formation [15]. Overexpression of the *kn4* gene in tomatoes results in a leaf curling and internode shortening phenotype, which can be observed in the transgenic plants through tissue sections containing less vascular tissue and smaller forming layer cells than in wild-type plants [16]. *KNAT2* belongs to the class I *KNOX* gene that hinders xylem differentiation and contributes to stem morphogenesis in poplar by regulating NAC domain transcription factors [17]. It has been demonstrated that *KNAT2* regulates the degree of leaf serration and is a vital gene involved in the morphogenesis [18].

Japanese apricot (*Prunus mume* Sieb. et Zucc.) is a Rosaceae (*Prunus* L.) fruit tree native to southwest China and has been extensively grown in East Asia and Japan for over 7000 years. Its fruit has significant medical and commercial value due to the high concentration of minerals and bioactive compounds. In this study, 11 Japanese apricot *KNOX* (*PmKNOX*) genes were identified, and their gene structure, chromosomal localization, and promoter cis-acting elements were analyzed. The KNAT2-related genes of the Japanese apricot were identified based on the KNOX family classification of peach (*Prunus persica*) because its gene functions have been explored. These results will contribute to further study into the functional properties of *PmKNOX* genes and provide a basis for elaborating the developmental functions of *PmKNOX* genes in leaves and stems.

## 2. Materials and Methods

### 2.1. Plant Materials

The samples of Japanese apricot were collected from the National Field GeneBank for *Prunus mume* in Nanjing, Jiangsu Province, China, with the cultivar ‘Longyan’ as the sampled cultivar. The root stems, leaves, flower buds, and leaf buds of Japanese apricot were collected in early October 2022, while fruit samples were collected in June of the same year. The samples were immediately put in liquid nitrogen and then transported and stored in a refrigerator at −80 °C for subsequent experiments.

Sterilized *Arabidopsis* seeds (Col0 and transgenes) were sown in 1/2MS medium. After vernalization at 4 °C, the seedlings were placed in a plant light incubator (photoperiod: 16 h light and 8 h dark; Constant temperature: 22 °C; Humidity: 80%) until four true leaves had developed. Seedlings with good growth traits were selected and transferred to the nutrient soil mixed with perlite and vermiculite for further experiments.

### 2.2. Identification of PmKNOX Family Members

The Japanese apricot genome (GCF_000346735.1_*P.mume*_V1.0) file and its annotation file were downloaded from NCBI (https://www.ncbi.nlm.nih.gov/, accessed on 20 October 2022). The hidden Markov model of KNOX (pfam03790, pfam03791, pfam03789 and pfam05920) was retrieved from PFAM (http://pfam.xfam.org/, accessed on 20 October 2022) as query domains for extracting KNOXs in *P.mume* local protein database. The candidate KNOXs (E value > 10–5) in Japanese apricot were screened using HMMER Software (http://hmmer.org/, accessed on 20 October 2022), and the KNOXs protein sequences were extracted by TBtools software [19]. After removing redundant and repeated sequences, the peach (*Prunus persica*) KNOX [20] was used as query sequences in a phylogenetic analysis with MAFFT v7.475 software [21]. We used the maximum likelihood (ML) method to construct phylogenetic trees to obtain the phylogenetic relationships. The ML analysis was conducted using IQ-TREE [22], with the best model selected by ModelFinder software [23]. The Bootstrap parameter is set to 1000 and beautified through iTOL software. Due to the location of KNOXs on the chromosome in *P. mume*, the genes were numbered PmKNOX1 to PmKNOX11. The protein properties of PmKNOXs, such as the molecular weight (MW), isoelectric point (pI) and grand average of hydropathicity (GRAVY) of each KNOX protein, were predicted by using ProtParam (https://web.expasy.org/protparam/, accessed on 21 October 2022). The BUSCA website (http://busca.biocomp.unibo.it/, accessed on 21 October 2022) was used to predict the subcellular localization.

### 2.3. Analysis of Structure and Conserved Motifs of PmKNOX Gene in Japanese Apricot

GSDS tool [24] was used to analyze and draw the gene structure map of *PmKNOX* gene family members. The protein sequences of *PmKNOX* genes were submitted to MEME (http://meme-suite.org/tools/meme, accessed on 22 October 2022) to search for conserved motifs of *PmKNOX*, setting the length to 6–50 and the number to 20 and other parameters were the default value and visualization of results by the software of TBtools.

### 2.4. Chromosomal Location and Syntenic Analysis

*P.mume*.gtf file and *PmKNOX* genes list was used as the input files, and TBtools software was used to analyze the location on the chromosomes of PmKNOX genes. Meanwhile, MCScanX software (http://chibba.pgml.uga.edu/mcsca n2/, accessed on 21 October 2022) was used to analyze the tandem and segmented replication events of PmKNOX family members.

### 2.5. Analysis of Cis-Acting Elements in PmKNOX Gene Upstream Promoter Region 

The 2000 bp region upstream of the *PmKNOX* transcription start site (ATG) was extracted from the *P. mume* genome, and the cis-acting elements were identified by the PlantCARE tool [25], and then these cis-acting elements were analyzed and classified. On the basis of the *PmKNOX* gene family sequence, a phylogenetic analysis was conducted, and protein sequences were aligned using the MAFFT software with default parameters [21]. ML was used to construct a phylogenetic tree to determine the phylogenetic relationship. The best model was chosen by ModelFinder software and used in the ML study using IQ-TREE. The iTOL programmer is used to enhance the Bootstrap parameter, which is set to 1000.

### 2.6. Expression Profiling Analyses of PmKNOX Genes

The *PmKNOX* gene expression pattern in the root, stem, leaf, leaf bud, flower bud, flower and fruit were also analyzed. Total RNA was extracted from tissue with an RNA extraction kit (Tiangen, China), and the concentration of extracted RNA was determined, and agarose gel electrophoresis was used to ensure its quality. For the synthesis of cDNA, it was performed in reference to the operation manual of PrimeScript™ RT reagent Kit with gDNA Eraser. qRT-PCR was performed using ABI 7300 real-time PCR system (Applied Biosystems, Foster, CA, USA) and SYBR green real-time PCR Master Mix (Dongbao, Osaka, Japan); primers are shown in Supplementray Table S1. Each bioreactor had three replicates in each analysis. The housekeeping Japanese apricot DNA-directed RNA polymerases II (*PmRP2,* LOC103335337) gene was determined as an internal control [26,27,28]. Three repeated quantitative analysis was performed for each cDNA sample, and the relative gene expression level was calculated by the 2^−∆∆CT^ method.

### 2.7. PmKNAT2a and PmKNAT2b Cloning and Sequence Analysis

The reference sequences of *PmKNOX2a* (LOC103343109) and *PmKNOX2b* (LOC103321797) were found in NCBI, and the Japanese apricot cv ‘Longyan’ was used as the template for gene cloning. Primers (Supplementary Table S1) were designed by reference sequence information, and cloning experiments were performed to obtain PCR products, which were subcloned into the clone007 blunt simple vector and sequenced at TsingKe (Nanjing, China). The sequences obtained were compared using BioXM 2.7 software.

### 2.8. Vector Construction, Plant Transformation, and Identification of Transgenic Lines

*PmKNAT2a* and *PmKNAT2b* coding sections were subcloned and placed into the p2301-35SN vector. The flower-dip technique was used to genetically modify *Arabidopsis* (by Agrobacterium-mediated means). Genomic DNA level detection, GUS staining confirmation, and RNA level detection were used to identify the transgenic lines.

### 2.9. Determination of Lignin Percentage Content

The stems of transgenic and wild-type plants at the same growth stage were placed in an oven at 80 °C, dried to constant weight and ground to powder. 3 mg of powder was extracted for lignin according to the instructions of Solarbio (Beijing, China) (BC4205 kit), and the absorbance value A at 280 nm was measured using a microplate reader (recording A as sample), and △A = A − A _control_ was calculated. The percentage (%) of lignin = ΔA ÷ ε ÷ d × V detected ÷ (V supernatant ×W ÷ V acetylated) ÷ 1000 × 100% (V acetylated: acetylation reaction volume, 0.612 mL. ε: lignin extinction coefficient, 23.35 mL/mg/cm. d: light diameter of cuvette, 1 cm. V supernatant: supernatant volume, 0.012 mL. V detection: detection volume, 0.6 mL. W: sample quality, g; 1000: conversion factor, 1 g = 1000 mg).

### 2.10. Measurement of Leaf Area

The original method of counting grids (1 mm^2^/grid) was used in this study to obtain more accurate leaf areas because the leaves margin of transgenic plants is curled and not well fixed. After tracing the leaf traits along the leaf margin on the grid paper, the number of grids was counted. Three strains with high expression of heterologous *PmKNAT2* were taken for the experiment, and three replicates were set up for each group of experiments.

### 2.11. Statistical Analyses

The student’s *t*-test was performed by IBM SPSS Statistics 23 programmer (ANOVA). To discover whether the test was significantly different or not, a 0.05 *p*-value cutoff was used. Using GraphPad Prism 8 software, standard deviation (SD) analysis of biological replicates of independent samples was performed, and error bars and graphs were plotted.

## 3. Results

### 3.1. Identification of KNOX Family Members in Japanese Apricot

**Figure 1:**
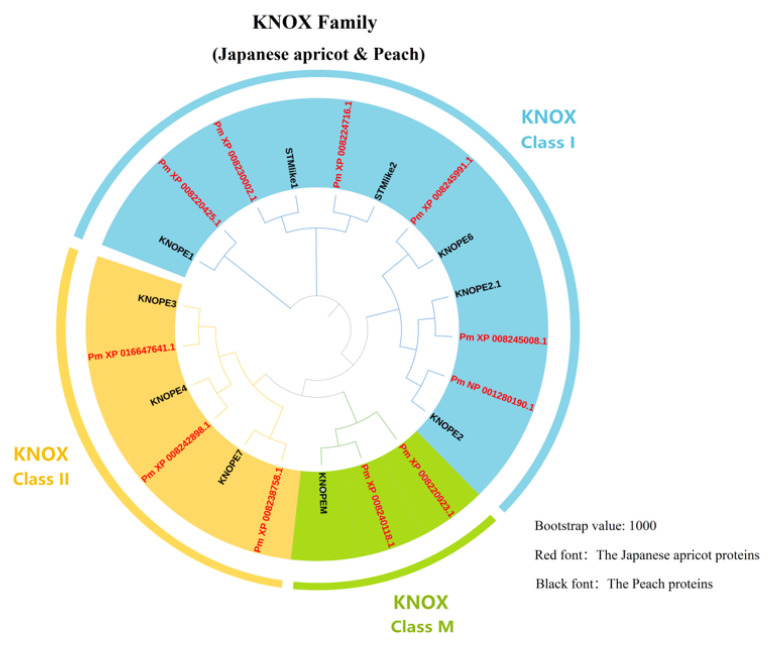
Phylogenetic tree of KNOX proteins from Japanese apricot and peach. The multiple protein sequences of 11 PmKNOX genes and 10 PpKNOX genes were aligned with the MUSCLE method, and the tree was built using the Maximum Likelihood method by using MAFFT v7.475 (bootstrap value: 1000). The tree was categorized into three subfamilies that include Class I (blue backdrop), Class II (yellow backdrop) and Class M (green backdrop). The PmKNOX proteins have been emphasized in red. The black font is the KNOX gene of peach.

The KNOX genes family conserved structural domains KNOX1 (pfam03790), KNOX2 (pfam03791), ELK (pfam03789) and Homeobox KN (pfam05920). A phylogenetic tree was constructed, including the candidate genes and ten KNOX genes from the peach (*Prunus persica*) (Figure 1) [29]. According to the division of Peach’s PpKNOX gene family [20], XP_008245991.1, XP_008220425.1, NP_001280190.1, XP_008224716.1, XP_008230002.1 and XP_008245008.1 belong to class I KNOX genes. XP_016647641.1, XP_008238758.1 and XP_008242898.1 belong to class II KNOX genes. XP_008220923.1 and XP_008240118.1 belonged to KNATM genes. According to the position of these genes on the chromosome, they are numbered PmKNOX1 to PmKNOX11 (Table 1).

The physical and chemical properties of *PmKNOX* family genes are shown in Table 1, including gene name, Gene ID, Protein ID, MW, pI, GRAVY value and subcellular location prediction. The average length of the CDS sequence of the *PmKNOX* gene is about 963 bp, of which the *PmKNOX2* (1293 bp) gene sequence is the longest, and the *PmKNOX9* (423 bp) sequence is the shortest.

The amino acid composition of PmKNOX family proteins is different, and their corresponding physical and chemical properties are also very different. The pI of the PmKNOX family was between 4.29 (PmKNOX4) and 6.53 (PmKNOX7). In general, all PmKNOX proteins are rich in acidic amino acids (Pi < 7) and are hydrophilic(GRAVY < 0). The prediction of the subcellular location of the PmKNOX gene family showed that all the PmKNOX genes were localized in the nucleus.

### 3.2. Secondary Structure Analysis of PmKNOX Protein

The analysis of the structure of the proteins encoded by the PmKNOX transcription factor family showed (Table 2) that there were some differences in the protein secondary structure among the members. The analysis of the protein secondary structure of protein allows us to determine the biological functions of PmKNOX proteins. Predictive analysis revealed that of the 11 PmKNOX protein sequences, six amino acid sequences (PmKNOX4, PmKNOX7, PmKNOX8, PmKNOX9, PmKNOX10 and PmKNOX11) were predominantly composed of α helix, and five amino acid sequences (PmKNOX1, PmKNOX2, PmKNOX3, PmKNOX5 and PmKNOX6) consisted mainly of random coils. β turn and Extended strands are scattered throughout each protein sequence.

### 3.3. Gene Structures, Protein Conserved Domain and Motif Compositions of PmKNOX Proteins

**Figure 2:**
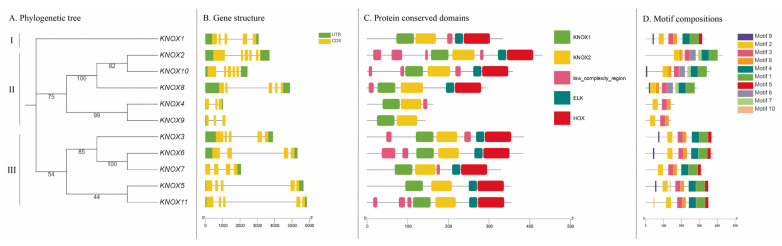
The conserved protein domains, gene structures and conserved motifs of *PmKNOX* genes are based on phylogenetic relationships. The analysis of *PmKNOX* (**A**) Phylogenetic tree (I, II and III mean the subfamily). (**B**) Gene structures. (**C**) Protein conserved domains. (**D**) Motif compositions. The multiple protein sequences of the 11 PmKNOX gene were aligned with the MUSCLE method, and the tree was built using the neighbor-joining method by using MEGA-X. The length of the gene structure and motif components can be estimated by referring to the corresponding scales below.

To investigate further the evolutionary relationships and gene structures among the *PmKNOX* members of Japanese apricot, we constructed a phylogenetic tree of the *PmKNOX* members (Figure 2A), mapped the *PmKNOX* gene structures (Figure 2B), and analyzed the characteristics of the conserved domains of the protein sequences and the composition of Motif (Figure 2C,D). The phylogenetic analysis showed that PmKNOX was divided into three subfamilies: *PmKNOX1* was an independent subfamily (Subfamily I); *PmKNOX2*, *PmKNOX10*, *PmKNOX8*, *PmKNOX4* and *PmKNOX9* were clustered into a subfamily (Subfamily II); *PmKNOX3*, *PmKNOX6*, *PmKNOX7*, *PmKNOX5* and *PmKNOX11* formed a subfamily (Subfamily III). This indicates that *PmKNOX1* may be genetically distant from the other *PmKNOX* genes. Structural analysis of the *PmKNOX* genes revealed that the number of exons in the *PmKNOX* genes ranged from 3 to 6, with *PmKNOX4* and *PmKNOX9* having three exons and *PmKNOX6* and *PmKNOX7* having four exons. The genes with the highest number of five exons were *PmKNOX1*, *PmKNOX10*, *PmKNOX8*, *PmKNOX3*, *PmKNOX5*, and *PmKNOX11*, and only one gene, *PmKNOX2*, had six exons. Most of the *PmKNOX* members have 5′UTR and 3′UTR, which contribute to the maintenance of mRNA stability and microRNA binding, with only *PmKNOX7* lacking the 5′UTR structure and *PmKNOX9* lacking the 3′UTR structure. The division of the *PmKNOX* subfamily was not significantly correlated with the structure of *PmKNOX* gene exons. However, *PmKNOX* genes classified into the same subfamily exhibited similar gene structure and Motif pattern.

As members of the *KNOX* gene family, all *PmKNOX* members contain the KNOX1 and KNOX2 conserved domains, whereas the majority of *PmKNOX* genes, except *PmKNOX4* and *PmKNOX9*, also contain the Homeobox_KN and ELK conserved domains. MEME analysis of the *PmKNOX* gene family composition of Motif revealed that all PmKNOX family members contained Motif2, Motif3 and Motif8. Except for PmKNOX4 and PmKNOX9 contained Motif1, and Motif4, whereas no subfamily II members contained the motif5 pattern. In addition, *PmKNOX10*, *PmKNOX8*, and *PmKNOX5* contained the largest number of Motifs, while *PmKNOX4* and *PmKNOX9* had only three Motifs. The above analysis indicates that the *PmKNOX* gene members have different gene functions among individuals. This is consistent with the previous description that class I and class II *KNOX* genes in angiosperms have different exon-intron structures [7].

### 3.4. Chromosomal Distributions Analysis of PmKNOX Genes

**Figure 3:**
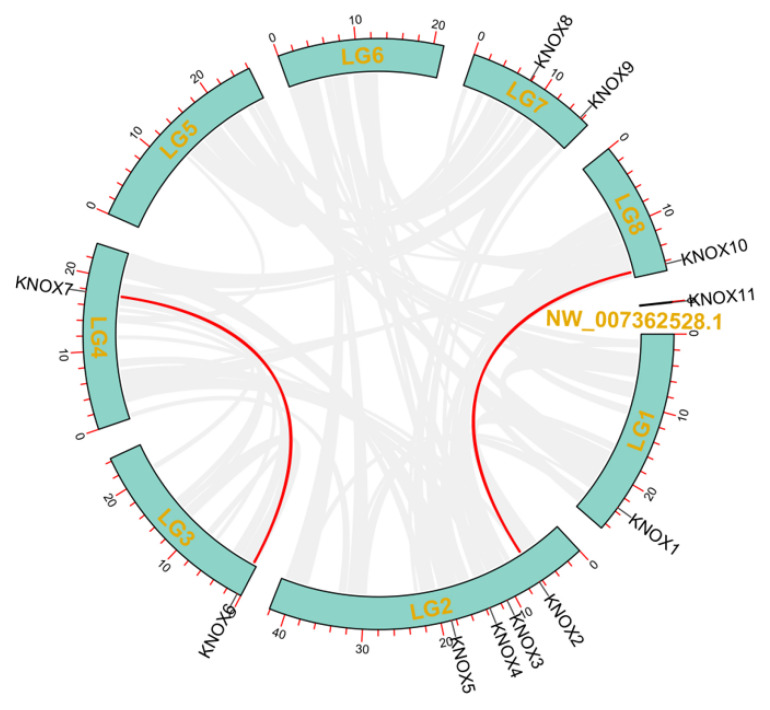
The distribution of the PmKNOX gene on the chromosomes and syntenic relationships. The scale on the left represents the length of chromosomes in megabases (Mb). Numbers are on each chromosome in yellow, and segmentally duplicated genes are linked with red lines.

Based on the annotation information of the Japanese apricot genome, the chromosome mapping of 11 *PmKNOX* genes was obtained (Figure 3). We found that the genes of *PmKNOX* were unevenly distributed on the chromosomes: *PmKNOX1* was located on LG1, *PmKNOX2*-*5* were all distributed on LG2, *PmKNOX6* and *PmKNOX7* were distributed on LG3 and LG4, respectively, *PmKNOX8* and *PmKNOX9* were distributed on LG7, *PmKNOX10* on LG8, and no clear position of *PmKNOX11* on the chromosome was obtained. On the Japanese apricot’s chromosomes LG5 and LG6, no members of the *PmKNOX* gene family were found (consistent with the results in Table 1). The analysis revealed that there was no significant correlation between the length of chromosomes and the distribution of *PmKNOX* genes. Colinear analysis of the *PmKNOX* genes revealed two pairs of segmental repeat genes (*PmKNOX2* and *PmKNOX10*, *PmKNOX6* and *PmKNOX7*).

### 3.5. Cis-Acting Elements Analysis in PmKNOX Gene Promoter Region

**Figure 4:**
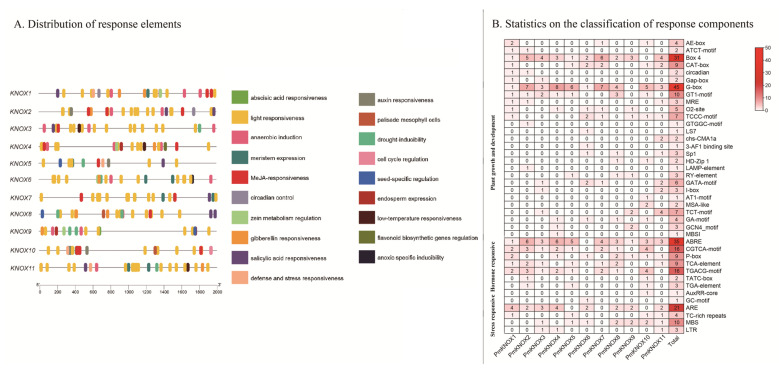
Predicted cis-elements in the *PmKNOX* promoters. (**A**) Analysis of the distribution of response elements on *PmKNOX* promoters. (**B**) Statistics on the classification and number of *PmKNOX* promoter response elements. The 2.0 kb sequence upstream from the coding sequence of *PmKNOX* genes was analyzed using the PlantCARE database.

In order to better understand the transcriptional mechanism of *PmKNOX*, we extracted and studied the 2000 bp promoter upstream of the coding sequence (CDS) of the eleven *PmKNOX* genes. The analysis revealed that the promoter region of *PmKNOX* genes contains a large number of cis-acting elements, which can be classified into three types: stress-responsive related elements type, hormone-responsive related elements type, and plant growth and development-related elements type (Figure 4).

Among all members of the *PmKNOX* gene family, the *PmKNOX2* gene contains 36 cis-acting elements, and all other genes contain more than 16 cis-acting elements. Analysis of the distribution of elements on the promoters of *PmKNOX* genes showed that there were 161 growth-related elements, of which 134 were related to light response, accounting for 83.2% of the elements. There are 10 meristem expression-related elements, accounting for 6%, and the rest are zein metabolism regulation elements, cell cycle regulation elements and flavonoid biosynthetic regulation elements, accounting for 10.8%. There were 91 hormone response-related elements, including 35 abscisic acid response elements, 32 methyl jasmonate response elements, 11 gibberellin response elements, 11 salicylic acid response elements, and 9 auxin response elements. 9 response elements, and 4 auxin response elements. There were 39 stress-related response elements, including anoxic specificity, anaerobic induction, drought-inducibility, low-temperature responsiveness, defense and stress. The above study indicates that *PmKNOX* genes are actively involved in plant growth and development and physiological and metabolic processes.

### 3.6. Expression Profiles Analysis of PmKNOX Genes in Different Tissues

**Figure 5:**
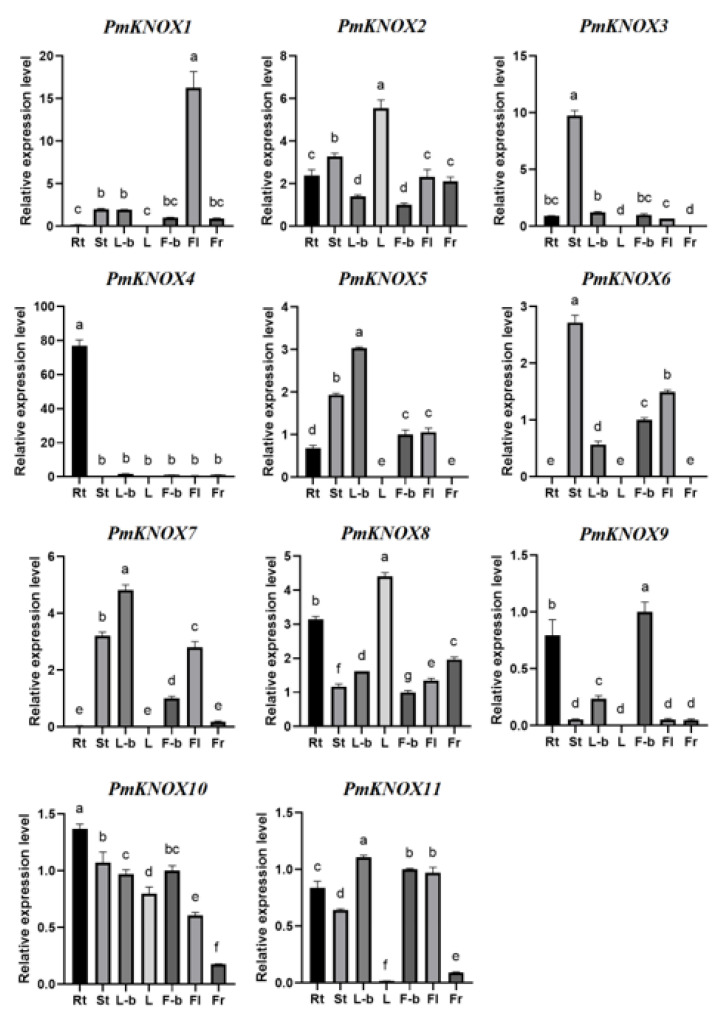
Expression analysis of the *PmKNOX* genes in different tissues. Rt for root, St for the stem, L for leaf, L-b for leaf bud, F-b for flower bud, Fl for flower and Fr for fruit. The letter indicates a significant change in the variance analysis (*p* < 0.05).

To investigate the expression pattern of *PmKNOX* genes in different tissues of Japanese apricot, the expression levels of 11 *PmKNOX* genes were analyzed in the root (Rt), stem (St), leaf (L), leaf bud (L-b), flower bud (F-b), flower (Fl) and fruit (Fr) using quantitative reverse transcription PCR technique (qRT-PCR) (Figure 5).

According to the findings, roots and leaves hardly ever expressed *PmKNOX1*, while flowers were the main organ. *PmKNOX3* was almost exclusively expressed in the stems and barely in the leaves and fruits. Roots had the highest levels of *PmKNOX4* expression compared to other tissues. *PmKNOX6* was almost entirely absent in roots, leaves, and fruits and was mostly expressed in stems and flowers. *PmKNOX7* was predominantly expressed in flower buds and leaf buds. The Japanese apricot’s *PmKNOX2*, *PmKNOX8*, and *PmKNOX10* genes were expressed in every tissue. These *PmKNOX* genes may be involved in the growth and development of Japanese apricots and may have various activities based on the varying expression levels of these genes in various tissues. The PmKNOX gene expression profile again showed that these genes were expressed at different levels in different tissues. Most of them were associated with the meristems of leaves and flower buds, suggesting that *PmKNOX* may be involved in the apical meristem of plants; these results support the previous conclusion that most *KNOX* family genes are important for maintaining the state of apical meristem [30,31,32]. In the phylogenetic tree created in conjunction with peach (*Prunus persica*) KNOX genes, PmKNOX5 and PmKNOX11 belonged to the same homeobox protein knotted-1-like 2 (Figure 1). *PmKNOX5* expression was significantly higher in leaf buds and stemmed than in other tissues, and *PmKNOX11* expression was also higher in leaf buds, indicating that these two genes may be crucial in the early stages of leaf and stem differentiation and merit further study.

### 3.7. Heterologous Expression of PmKNAT2 Genes in Arabidopsis

**Figure 6:**
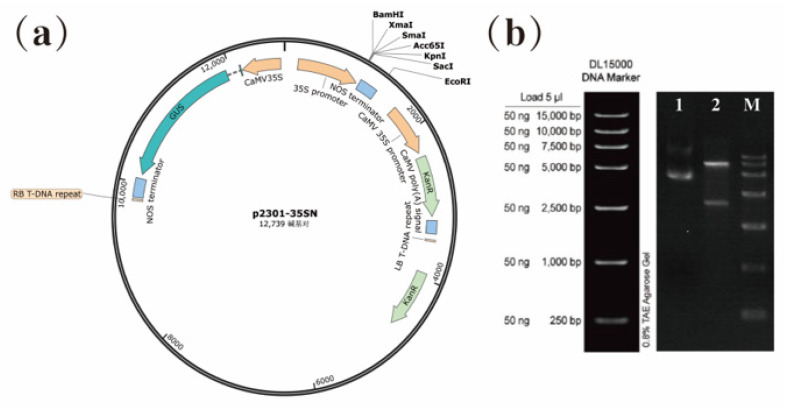
Construction of expression vector of *PmKNAT2-a* and *PmKNAT2-b*. (**a**) Vector mapping of p2301-35SN; (**b**) Construction of p2301-*PmKNAT-a* vector. M is Marker; lane 1 is p2301-*PmKNAT2/6-a* vector plasmid; lane 2 is enzyme digestion assay (restriction enzyme: *BgllI*).

Since *PmKNOX5* and *PmKNOX11* are more closely related to *PpKNAT2* of peach (*Prunus persica*) and belong to the *Homeobox Knotted-1-like 2*, these two genes were named *PmKNAT2a* (formerly *PmKNOX11*) and *PmKNAT2b* (formerly *PmKNOX5*) for simplicity for subsequent studies and classification. The CDS of *PmKNAT2-a* and *PmKNAT2-b* were obtained by cloning, respectively, and the CDS of these two genes were subcloned into the expression vector p2301-35SN (Figure 6).

**Figure 7:**
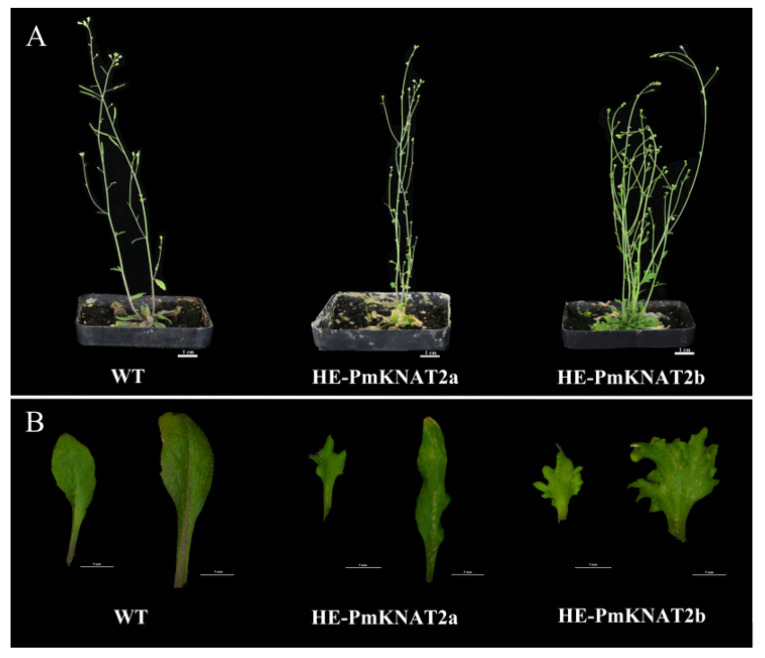
Phenotypic analysis of HE-PmKNAT2a and HE-PmKNAT2b. (**A**) Comparison of T1-generation transgenic plants with the highest heterologous expression and wild-type *Arabidopsis*. (**B**) Comparison of basal leaves of transgenic plants (left) and leaves on the main stem near the base (right) with WT plants. WT represents wild-type *Arabidopsis*, and HE-PmKNAT2a and HE-PmKNAT2b represent transgenic plants heterologously expressing the *PmKNAT2a* and *PmKNAT2b* genes in *Arabidopsis*, respectively.

The vector plasmids were transformed into *Agrobacterium tumefaciens* and then infested with *Arabidopsis*. Four heterologous expressions of *PmKNAT2-a Arabidopsis* lines (HE-PmKNAT2-a) and five heterologous expressions of *PmKNAT2-b Arabidopsis* lines (HE-PmKNAT2/6-b) were obtained by screening the transgenic strains (Supplementary Figure S1). Phenotypic analysis revealed that HE-PmKNAT2a and HE-PmKNOX2b *Arabidopsis* leaves all became lobed (more pronounced in HE-PmKNOX2b) with curled leaf margins compared to the wild type (Figure 7). This is consistent with the phenotype of *Arabidopsis* heterologously transformed with *KNAT2* from maize and *Pinus pinaster* [14,33,34].

In comparison with the wild type, we found an interesting phenomenon: heterologous expression of *PmKNAT2a* resulted in smaller leaf area near the base and basal leaves in *Arabidopsis*, whereas heterologous expression of *PmKNAT2b* resulted in larger leaves at the base and smaller leaves near the base in *Arabidopsis* (Table 3).

### 3.8. Effect of Heterologous Expression of PmKNAT2a and PmKNAT2b on Arabidopsis Stems

**Figure 8:**
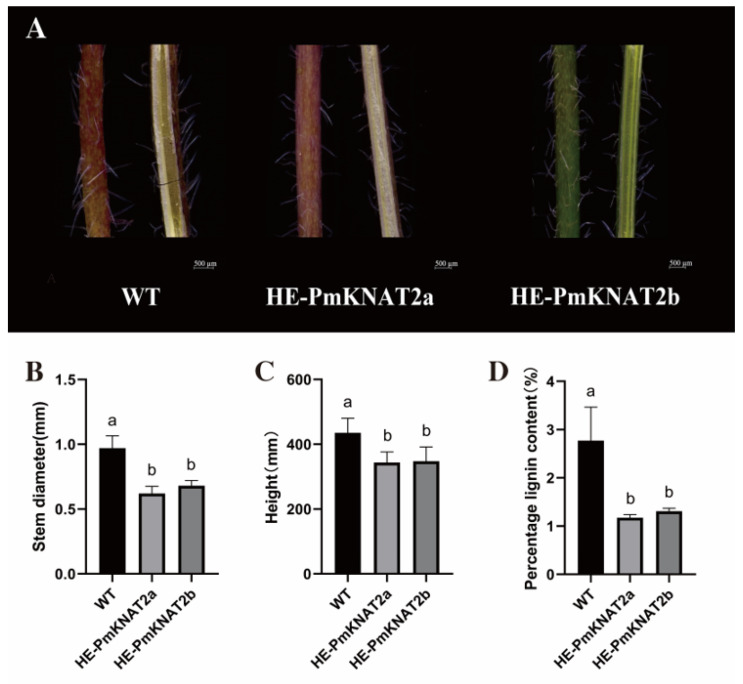
Effects of HE-PmKNAT2a and HE-PmKNAT2b on stems. (**A**) Comparison of longitudinal stem sections of HE-PmKNAT2a, HE-PmKNAT2b and WT. (**B**) Comparison of the stem diameters of HE-PmKNAT2a, HE-PmKNAT2b and WT. (**C**) Comparison of plant height of HE-PmKNAT2a, HE-PmKNAT2b and WT. (**D**) Comparison of the lignin content percent of HE-PmKNAT2a, HE-PmKNAT2b and WT. WT represents wild-type *Arabidopsis*, and HE-PmKNAT2a and HE-PmKNAT2b represent transgenic plants heterologously expressing the *PmKNAT2a* and *PmKNAT2b* genes in *Arabidopsis*, respectively. The letter indicates a significant change in the variance analysis (*p* < 0.05).

To investigate the effects of heterologous expression of *PmKNAT2a* (HE-PmKNAT2a) and *PmKNAT2b* (HE-PmKNAT2b) on the growth of *Arabidopsis* stems, we intercepted the stems of HE-PmKNAT2a, HE-PmKNAT2b, and wild-type plants (WT) near the base, respectively, and showed their stem phenotype (Figure 8A). By observation, we found that the WT and the HE-PmKNAT2a stems exhibited a red color, while the HE-PmKNAT2b stems were green. Analysis of the leaf spacing of HE-PmKNAT2a and HE-PmKNAT2b revealed (Table 4) that the leaf spacing of HE-PmKNAT2a was shorter than that of the wild type, and HE-PmKNAT2b was similar to the wild type only between the distance from the base to the first leaf, while all other leaf spacing was shorter than that of the wild type. The measurement of stem diameter revealed that the stems of wild-type *Arabidopsis* plants were significantly thicker than those of transgenic *Arabidopsis* (Figure 8B). Meanwhile, the plant height was measured, and it was found that the plants of wild-type *Arabidopsis* were significantly longer than the transgenic type (Figure 8C). A similar phenotype was observed in *Populus* overexpressing *Populus alba* × *P. glandulosa KNAT2/6b* (*PagKNAT2/6b*) [35].

Since the class I *KNOX* genes have been shown to be involved in the lignin deposition process [36,37]. Therefore we tested the lignin percentage content of transgenic and wild-type *Arabidopsis* stems (Figure 8D). It was discovered that lignin concentration was much reduced in plants with HE-PmKNAT2a and HE-PmKNAT2b compared to the wild type, indicating that *PmKNAT2a* and *PmKNAT2b* may be involved in the process of lignin deposition in plants.

## 4. Discussion

The *Knotted1-like Homeobox* (*KNOX*) genes are important regulators of meristem function, and their expression is tightly controlled by a complex network of transcription factors [38,39]. In the present study, 11 *KNOX* genes members have been identified in the genome of the Japanese apricot, with almost the same number as in tomato (8 members) [40], *Arabidopsis* (9 members) [11], coastal pine (6 members) [33], but less than apple (22 members) [4], Soybean (34 members) [3], etc. This result indicates that most *PmKNOX* genes have not been selectively eliminated by the environment, and no obvious gene family amplification events have occurred.

Phylogenetic analysis of KNOX proteins in Japanese apricot and *Arabidopsis* revealed that the Japanese apricot KNOX family is divided into three subfamilies: class I *KNOX* genes, class II *KNOX* genes and class M *KNOX* genes. The class I *KNOX* genes *KNAT1*, *KNAT2* and *KNAT6* in *Arabidopsis* are expressed in the flower meristem and affects the process of flower development [41,42]. The class I *KNOX* genes are specific in temporal and spatial expression patterns, have both redundant functions and their own specific effects in function, and eventually affect the morphogenesis of lateral organs by regulating cell differentiation [7,43]. The class II KNOX proteins operate as repressors in the transcriptional control of their target genes, in contrast to class I KNOX proteins, which appear to be activators [44]. The Class II *KNOX* genes include *KNAT3, KNAT4*, *KNAT5*, and *KNAT7*, which are expressed in all plant tissues and are associated with seed germination and secondary wall formation [45]. The class M *KNOX* genes are mainly expressed at the boundary between plant organ primordium and mature organs and may be involved in sensing or generating flowering signals [11]. Thus, the potential function and research direction of Japanese apricot *KNOX* clustered into the same group of *KNOX* genes in *Arabidopsis* can be inferred.

Combined gene structure and conserved Motif analysis revealed that *PmKNOX* belonging to the same subfamily exhibit similar gene structure and motif patterns, which may exercise similar biological functions. In general, the differences in the exon and intron structure of genes are the main reasons for the diversification of gene functions. The *PmKNOX* genes show similar intronic and exonic structures within the same subfamily, indicating high conservation of the family members in evolution, and the results were consistent with those of previous studies [7]. The predicted protein secondary structures of the 11 sequences showed that the PmKNOX protein sequence was mostly made up of random coils or α helices. Chromosome mapping results showed that 11 *PmKNOX* members were distributed in 6 chromosomes, and two pairs of genes *PmKNOX* genes had a collinear relationship.

The predicted cis-acting elements of *PmKNOX* genes showed that the promoter regions of these 11 *PmKNOX* gene family members were rich in a large number of growth and development-related elements, hormone response-related cis-acting elements and stress-related elements. Therefore, the *PmKNOX* gene may affect plant growth, development and morphogenesis by influencing hormonal and metabolic pathways. According to RT-qPCR analysis, like in other plants, the majority of the *PmKNOX* family genes were slightly expressed differently in various tissues [46], indicating that *PmKNOX* is probably actively involved in a variety of plant growth and developmental processes [7,47]. At the same time, we noticed that *PmKNOX* was highly expressed in the leaf buds and flower buds of Japanese apricot, indicating that *PmKNOX* played an important role in maintaining the meristem potential, which is consistent with previous research findings [14,48].

*PmKNOX5* and *PmKNOX11* are phylogenetically related to the class I *KNOX* gene *KNAT2* of peach (Figure 1). Additionally, *KNAT2* has been studied more intensively in many species, and several studies have shown that this class of genes regulates the formation and growth of lateral organs and can be expressed in the apical meristematic tissue to determine the fate of meristematic cells, which plays an important role in leaves, stems, etc. of plants [18,33,42,49]. However, functional studies on the *PmKNAT2* gene in Japanese apricot have not been conducted. To perform a functional analysis of the *PmKNAT2* gene of Japanese apricot in *Arabidopsis*, *PmKNOX11* was named *PmKNAT2a* and *PmKNOX5* named *PmKNAT2b* based on phylogenetic relationships, and the phenotypes of transgenic *Arabidopsis* observed. Leaf shape in plants is regulated by genetic, developmental, and environmental factors involving the regulation of a complex genetic network between hormonal signals and transcription factors. In *Arabidopsis,* leaves heterologously expressing *PmKNAT2a* (HE-PmKNAT2a) and *PmKNAT2b* (HE-PmKNAT2b) had altered leaf phenotypes. Leaves of HE-PmKNAT2ah and HE-PmKNAT2b were twisted with lobed leaf margins, with HE-PmKNAT2b having more pronounced lobbing. It has been shown that leaves with deeper serrations or fissures have shorter heat transfer distances compared to full-rimmed leaves and are able to increase heat dissipation efficiency per unit leaf area, which has an important role in plant response to high-temperature stress [50].

In leaf area determination, heterologous expression of *PmKNAT2a* resulted in smaller leaf area near the base and basal in *Arabidopsis*, whereas heterologous expression of *PmKNAT2b* resulted in larger leaves near the base and smaller leaves near the base in *Arabidopsis*. It has been shown that *REDUCED COMPLEXITY* (*RCO*) and class I *KNOX* genes in *Cardamine hirsuta* leaves increase leaf complexity (i.e., there will be various morphological and size-invariant leaves), similar to the findings herein, and demonstrates that *PmKNAT2* may have a function in regulating leaf development. In addition, these two genes are barely expressed in leaves, suggesting that the period during which they are involved in leaf morphogenesis should be the period of leaf bud differentiation. These results seem to confirm the finding that class I *KNOX* genes regulate plant morphogenesis [51].

Lignin is commonly found in the xylem, parenchyma and cortical cells and is one of the main components of the xylem, and its biosynthesis plays an important role in plant cell growth and differentiation [52]. The class I *KNOX* genes may indirectly affect lignin synthesis by regulating the synthesis and metabolism of GAs or directly regulate the expression of lignin-synthesizing genes that affect the lignin deposition [53]. Combining these phenotypes of the phenomenon of shorter, thinner stems, shorter leaf spacing, and lower lignin content in HE-PmKNAT2a and HE-PmKNAT2b suggests that this may be related to class I *KNOX* gene expression promoting GAs synthesis [54]. *GA20ox*, a key enzyme in the plant GAs degradation process, is mainly expressed in the xylem and overexpression in poplar leads to stem elongation of the plant [55]. *GA2ox*, a key enzyme in plant GAs synthesis, is overexpressed in persimmon (*Diospyros kaki*) and leads to dwarfism of the plant [56]. These two genes work together in the plant to maintain the dynamic balance of active GAs. It has been demonstrated that class I *KNOX* genes in *Rorippa aquatica*, potato (*Solanum tuberosum*), and maize apical meristem tissues can directly bind to the active element on the *GA20ox* gene promoter, thereby inhibiting the expression of *GA20ox* and leading to dwarfing of the plant [57,58,59]. The *PagKNAT2/6b* gene of poplar is a homolog of *Arabidopsis KNAT2* and *KNAT6*, and when this gene was overexpressed, the internode elongation of poplar was found to be inhibited, the content of GAs was significantly decreased, and anatomical analysis revealed that the length of epidermal cells, medullary cells, xylem fibers and ductal cells in transgenic poplar stems were significantly shorter [17]. Overexpression of class I *KNOX* genes in *Arabidopsis* leads to dwarf plants and leaf ruffling, and the aforementioned phenotypes make transgenic plants more drought tolerant, while studies have demonstrated that class I *KNOX* responds to drought-stressed environments by mediating the ability of stem cell division and differentiation in phloem tissues [60]. In this study, a preliminary exploration of the gene function of *PmKNAT2* was conducted in *Arabidopsis* heterologously transformed with the *PmKNAT2* gene, which was found to be potentially involved in leaf and stem development and lignin biosynthesis.

## 5. Conclusions

This study identified 11 *KNOX* gene family members on six chromosomes of the Japanese apricot genome and that PmKNOX can be divided into three subfamilies in phylogenetic tree analysis with KNOX proteins in *Arabidopsis* (I, II and M). The 11 *PmKNOX* gene structure and conserved Motif analysis showed that *PmKNOX* from other same subfamily had similar gene structure and motif patterns, thus suggesting that they have similar biological functions. The promoter analysis and expression profile analysis of the *PmKNOX* gene showed that it was actively involved in physiological metabolism, growth and development and may play a potential role in the apical meristem. Heterologous expression of *PmKNAT2a* and *PmKNAT2b* resulted in shorter plant height and abnormal leaf edge development in *Arabidopsis* and decreased lignin in stems. Hence these two genes may regulate leaf and stem development. This research advances our knowledge of the origin and biological significance of the KNOX gene family in the Japanese apricot and provides a theoretical foundation for the future application of *PmKNOX* functional gene studies.

## Figures and Tables

**Table 1:** Identification of the *KNOX* gene family in the Japanese apricot genome.

Name	Gene ID	Protein ID	Chromosomal Position	gDNA (bp)	CDS (bp)	Protein				
						Length (aa)	MW (kDa)	pI	GRAVY	Subcellular Localization Prediction
*PmKNOX1*	LOC103344141	XP_008245991.1	LG1: 23,453,520–23,456,592	3073	1002	333	37.683	5.06	−0.67	nucleus
*PmKNOX2*	LOC103319886	XP_016647641.1	LG2: 6,437,754–6,441,461	3708	1293	430	undefined	undefined	−0.844	nucleus
*PmKNOX3*	LOC103320515	XP_008220425.1	LG2: 11,142,302–11,146,201	3900	1161	386	44.011	6.13	−1.011	nucleus
*PmKNOX4*	LOC103320960	XP_008220923.1	LG2: 13,532,590–13,533,609	1020	489	162	17.988	4.29	−0.583	nucleus
*PmKNOX5*	LOC103321797	NP_001280190.1	LG2: 18,638,472–18,644,136	5665	1062	353	40.407	5.09	−0.727	nucleus
*PmKNOX6*	LOC103324440	XP_008224716.1	LG3: 521,722–527,036	5315	1152	383	42.812	6.3	−0.738	nucleus
*PmKNOX7*	LOC103329326	XP_008230002.1	LG4: 17,650,120–17,652,180	2061	990	329	36.901	6.53	−0.63	nucleus
*PmKNOX8*	LOC103337382	XP_008238758.1	LG7: 7,825,616–7,830,499	4884	877	291	33.059	6.35	−0.73	nucleus
*PmKNOX9*	LOC103338667	XP_008240118.1	LG7: 15,732,193–15,733,759	1567	423	140	15.732	4.81	−0.759	nucleus
*PmKNOX10*	LOC103341196	XP_008242898.1	LG8: 16,386,362–16,389,098	2737	1077	358	40.833	5.61	−0.795	nucleus
*PmKNOX11*	LOC103343109	XP_008245008.1	Unplaced Scaffold: 110,247–116,106	5860	1066	354	39.674	5.45	−0.667	nucleus

**Table 2:** Analysis of the secondary structure of PmKNOX protein.

Protein Name	α Helix	Extended Strand	β Turn	Random Coil
PmKNOX1	149 (44.74%)	19 (5.71%)	13 (3.90%)	152 (45.65%)
PmKNOX2	178 (41.40%)	24 (5.58%)	7 (1.63%)	221 (51.40%)
PmKNOX3	155 (40.16%)	30 (7.77%)	12 (3.11%)	189 (48.96%)
PmKNOX4	95 (58.64%)	14 (8.64%)	5 (3.09%)	48 (29.63%)
PmKNOX5	154 (43.63%)	11 (3.12%)	14 (3.97%)	174 (49.29%)
PmKNOX6	147 (38.38%)	41 (10.70%)	20 (5.22%)	175 (45.69%)
PmKNOX7	176 (53.50%)	14 (4.26%)	13 (3.95%)	126 (38.30%)
PmKNOX8	168 (57.73%)	8 (2.75%)	10 (3.44%)	105 (36.08%)
PmKNOX9	98 (68.53%)	2 (1.40%)	1 (0.70%)	42 (29.37%)
PmKNOX10	191 (53.35%)	11 (3.07%)	7 (1.96%)	149 (41.62%)
PmKNOX11	180 (50.85%)	11 (3.11%)	24 (6.78%)	139 (39.27%)

**Table 3:** HE-PmKNAT2 *Arabidopsis* leaf area compared with wild type.

Sample Name	Leaf Area of Basal Leaves/cm^2^	Leaf Area of Leaves near the Base/cm^2^
WT	1.22 ± 0.35	0.82 ± 0.08
HE-PmKNAT2a	0.65 ± 0.31	0.39 ± 0.06
HE-PmKNAT2b	1.14 ± 0.31	0.59 ± 0.20

**Table 4:** Leaf spacing in HE-PmKNAT2 *Arabidopsis* compared to wild type.

Sample Name	0~1/cm	1~2/cm	2~3/cm
WT	2.24 ± 0.08	2.06 ± 0.13	1.43 ± 0.09
HE-PmKNAT2a	1.24 ± 0.1	1.38 ± 0.22	1.24 ± 0.08
HE-PmKNAT2b	2.34 ± 0.24	1.46 ± 0.18	1.37 ± 0.14

Note: 0~1 represents the length from the base to the first leaf, 1~2 represents the length of the first leaf to the second leaf, and 2~3 represents the length of the second leaf to the third leaf.

## Data Availability

Not applicable.

## Supplementary Materials

The following supporting information can be downloaded at: https://www.mdpi.com/article/10.3390/genes14040939/s1, Table S1: Primer sequences used for qRT–PCR and PCR in Japanese apricot; Figure S1: Molecular identification of heterologous expression of *PmKNAT2-a* and *KNAT2-b* in *Arabidopsis*.
