# Supplementary material for: Genome-Wide Identification of the KNOX Gene Family in Japanese Apricot (Prunus mume Sieb. et Zucc.) and Functional Characterization of PmKNAT2 Genes
Source: Genes (Basel). 2023 Apr 18;14(4):939. doi: 10.3390/genes14040939 (PMC10138190; doi:10.3390/genes14040939)
Supplement: Supplementary file 1 [file genes-14-00939-s001.zip › genes-2286554-supplementary.pdf]

Table S1. Primer sequences used for qRT–PCR and PCR in Japanese apricot.

Genes	Sequences (5′-3′)	
	Forward	Reverse
PmKNOX1	AACGGAGGGCATGGAATCAG	TGGTTGCTTCATCGAACGGT
PmKNOX2	AAGCATGAGCTGAAGCAGGG	GCGTTTGCTCTTCAAAACTGT
PmKNOX3	ATCAAGAGGGCACCAACCAG	AAGCCTCTCTTGAAGCCACC
PmKNOX4	AACTGTTTGACGGTGGGGTT	CTCAAGTGGAGGTGTACGGG
PmKNOX5	ACTTCATCTGCTCACGCCTC	CAAGGTGCCAATGTGACTGC
PmKNOX6	CAGCTGCCGCCTGTTATTTC	ATCCGCTGCTGCTACTCATC
PmKNOX7	CAGCTCCAAGCTCTCACAGT	GGCCACTTGTAGTGTCTGCT
PmKNOX8	ATGTCAGAGTCCATGCCGTG	ATCCCATCATGTCATGCCCG
PmKNOX9	TGACCTAGACCTGGAGGGAG	GGTCAACAGACTGACCGGAA
PmKNOX10	AAGACGAACTGGAGTGCGAG	ACATCCCCATTGGCTCGAAG
PmKNOX11	TGGGAGAAGAGGTTTCGTGC	AAGCATCGAGCTCAGGATCG
PmRP2	TGAAGCATACACCTATGATGATGAAG	CTTTGACAGCACCAGTAGATTCC
PmKNAT2a-CDS	ATGATGGAGGAACTGTACGGC	TCAGTCCTTGGTAAAACAAGG
PmKNAT2b-CDS	ATGGAGGAAATGTACGGATTGC	TCAGTCATCTGTGAAAAATGG

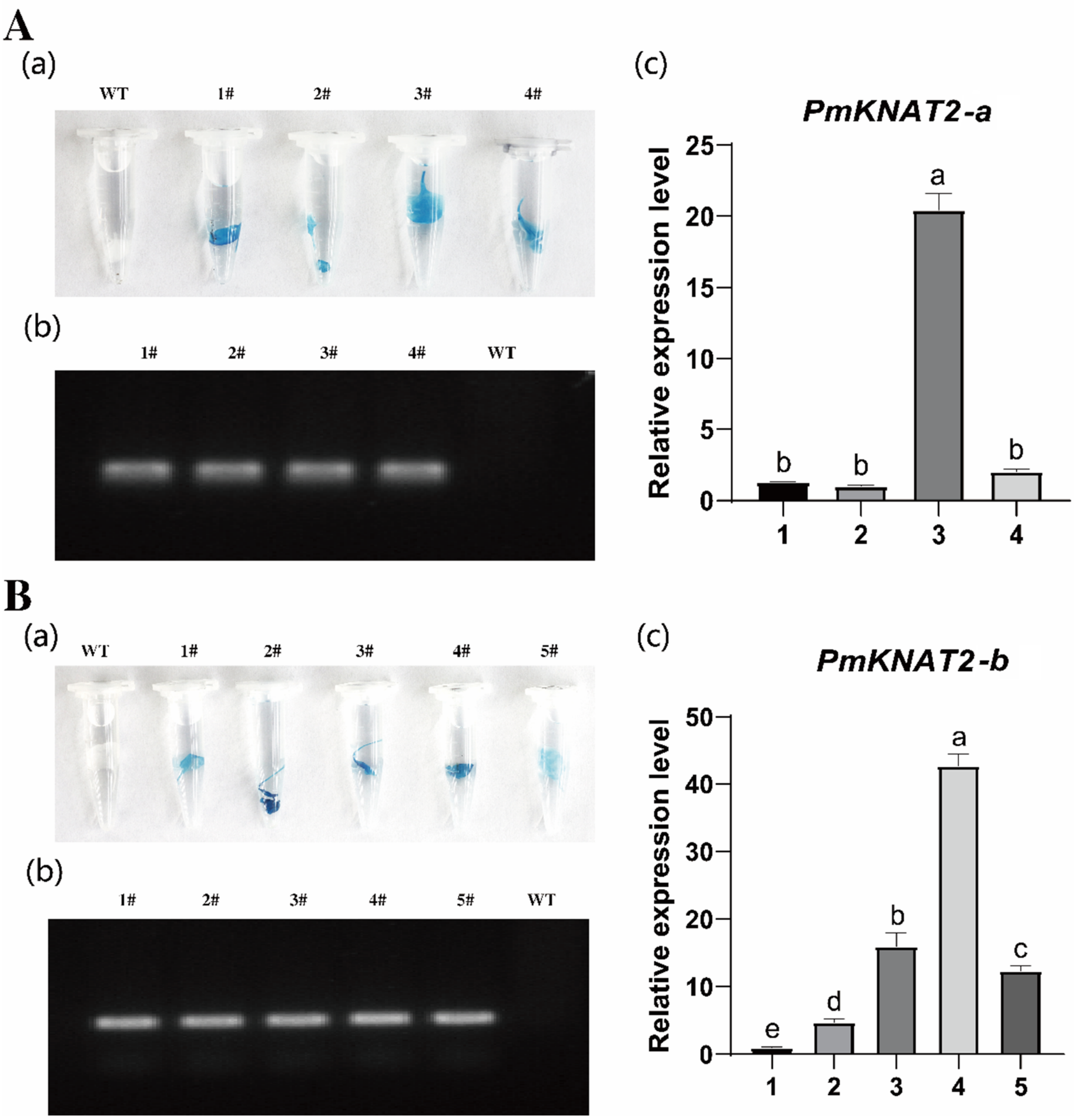

Figure S1. Molecular identification of heterologous expression of *PmKNAT2-a* and *KNAT2-b* in *Arabidopsis*. (A) GUS staining (a), detection of target gene insertion into the genome(b) and expression of *PmKNAT2*-a in transgenic lines (c). B. GUS staining (a), detection of target gene insertion into the genome(b) and expression of *PmKNAT2-b* in transgenic lines (c).
